# Interplay between family history and polygenic risk for coronary heart disease: A cohort study among over 60 thousand individuals

**DOI:** 10.1016/j.ajpc.2026.101497

**Published:** 2026-02-22

**Authors:** Carlos Iribarren, Meng Lu, Roberto Elosua, Martha Gulati, Nathan D. Wong, Jamal S. Rana

**Affiliations:** a Kaiser Permanente Northern California Division of Research, Pleasanton, CA, USA; b Cardiovascular Epidemiology and Genetics, Hospital del Mar Research Institute, Barcelona, Spain; c CIBER Cardiovascular Diseases (CIBERCV), Barcelona, Spain; d Faculty of Medicine, University of Vic-Central University of Catalonia (UVic-UCC), Vic, Spain; e Davis Women's Heart Center, Department of Cardiology, Houston Methodist DeBakey Heart & Vascular Center, Houston, TX, USA; f Heart Disease Prevention Program, Mary and Steve Wen Cardiovascular Division, Department of Medicine, University of California Irvine, Irvine, CA, USA; g Department of Cardiology, The Permanente Medical Group, Kaiser Permanente Oakland Medical Center, Oakland, CA, USA

**Keywords:** Genetics, Polygenic risk score, Family history of CAD

The heritability of coronary heart disease (CHD) is well established [1,2] and therefore family history of CHD is a recognized risk factor [3]. Family history is low cost and non-invasive and captures both genetic and non-genetic familial risk. However, it is considered to have low accuracy due to recall bias, and its definition can vary depending on inclusion of all first-degree relatives (parents plus siblings) or parents only.

Polygenic risk scores (PRS) have proven clinical utility alongside established clinical risk assessment tools [4]. However, few studies have systematically evaluated the overlap of family history and polygenic risk as well as their contributions to future CHD risk when both factors are considered in the risk prediction model.

The aim of this study was to ascertain the degree to which polygenic risk adds to self-reporting of family history of coronary heart disease (CHD) risk in a large, multiethnic real-world cohort in Northern California, USA, using a commercially available CHD PRS.

This study made use of genome-wide genetic data obtained from the Genetic Epidemiology Research on Aging (GERA) cohort of adult members of Kaiser Permanente of Northern California (KPNC). Recruitment, data collection, genotyping and outcome ascertainment through 12/31/2022 has been published [4]. Family history of CHD was ascertained via questionnaire by eliciting whether a first degree relative had ever suffered angina pectoris or heart attack (however no age at event of the relative was obtained). The study was approved by the Kaiser Foundation Research Institute Institutional Review Board, and all individuals provided informed consent.

The initial GERA cohort comprised 110,226 individuals recruited in 2007–08. Sequential baseline exclusions included incomplete genetic data for estimation of the PRS and/or principal components (PC) of genetic ancestry (*n* = 12,293), age 〈 30 or 〉 74 years (*n* = 8416), prevalent CHD (*n* = 2610), missing covariates (body mass index [BMI], LDL-C, smoking status, diabetes, hypertension; *n* = 23,877), and missing family history of CHD (*n* = 1678). The final cohort comprised 61,352 individuals. After a mean (SD) follow-up time of 13.8 (3.8) years, 3040 incident CHD events (including myocardial infarction, coronary revascularization procedures, angina pectoris, or CHD death) occurred. Further details of the PRS (CARDIO inCode-Score® CHD PRS, GENinCode Plc) description and validation can be found elsewhere [4]. Regarding the number of genetic variants included in our PRS, our rationale was to incorporate variants independently associated with CHD and not related to classical cardiovascular risk factors, with the aim of improving the predictive performance of cardiovascular risk functions. In a previous study in which we analyzed a PRS comprising >50 variants, the results were comparable to those obtained with the 12-variant PRS [5].

We estimated the proportions reporting family history of CHD according to quintiles of PRS and age-adjusted rates per 10,000 person-years of incident CHD by joint categories of PRS and family history of CHD (yes/no) in each of the PRS groups (low, first quintile; intermediate, quintiles 2–4; high, fifth quintile) using Poisson regression.

We then performed a series of multivariable Cox proportional hazards models, first considering main effects of PRS and family history, with adjustment for covariates (age, sex, 10 principal components of genetic ancestry, education level, smoking status, BMI, hypertension, diabetes, total cholesterol/HDL ratio and use of cholesterol lowering medications). Second, we constructed joint effects of PRS and family history (10 categorical variables), with first quintile and no family history as the reference group. Lastly, we modelled the effect of PRS separately in those with and without family history and tested the statistical multiplicative interaction between PRS (as a continuous variable) and family history as a dichotomous variable.

At baseline, the cohort had a mean (SD) age of 58 (10) years, and 62 % of the participants were female. Approximately 82 % self-identified as European, 3 % as African-American, 7 % as Latino, and 8 % as Asian.

Overall, 30.0 % reported family history of CHD, this proportion increased gradually from 27.2 % in quintile 1 of the PRS to 32.3 % in quintile 5 of the PRS (p-trend <0.0001). Age-adjusted rates of incident CHD increased steadily with increasing polygenic risk in both groups of family history of CHD (p-trend<0.0001) (Fig. 1 Panel A). Absolute rates (%) ranged from 3.5 % in the PRS quintile 1/No family history of CHD group to 7.8 % in the PRS quintile 5/Yes family history of CHD group. In the fully-adjusted main effects model there were statistically significant independent effects of PRS (relative to quintile 1) in quintiles 2–4 (hazard ratio [HR]=1.27; 95 % CI, 1.16–1.40; *p* < 0.0001), PRS in quintile 5 (HR=1.64; 95 % CI, 1.47–1.84; *p* < 0.0001) and family history of CHD (HR=1.42; 95 % CI, 1.32–1.53; *p* < 0.0001).

**Fig. 1:**
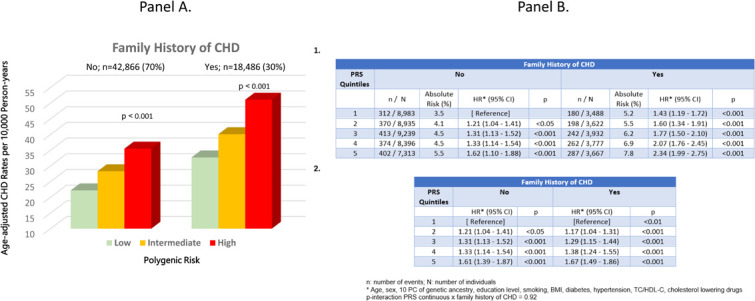
**Panel A**. Age-adjusted CHD rates per-10,000 person-years in joint categories of PRS and family history of CHD. **Panel B. 1**. Adjusted hazard ratios (95 % CI) of CHD by joint categories of family history of CHD and polygenic risk in the GERA cohort (*n* = 61,352). **2**. Adjusted hazard ratios (95 % CI) of CHD associated with polygenic risk stratifying by family history of CHD in the GERA cohort (*n* = 61,352).

In the model with joint effects of PRS and family history with a single reference level (i.e., no family history and PRS in quintile 1), the combination of positive family history of CHD and high polygenic risk conferred a HR of 2.34 (95 % CI 1.99–2.75); *p* < 0.0001) (Fig. 1 Panel B.1). In the model with stratification by family history of CHD, where in each group PRS in quintile 1 was the reference level, PRS is quintile 5 had very similar strength of association regardless of family history: the HR was 1.61 (95 % CI, 1.39–1.87; *p* < 0.0001) among those with no family history of CHD and 1.67 (95 % CI, 1.49–1.86; *p* < 0.0001) among those with family history of CHD (Fig. 1 Panel B.2). In agreement with these risk estimates, the interaction between PRS (as a continuous variable) and family history (as a categorical variable) was not statistically significant (*p* = 0.92). However, the lack of statistical interaction does not necessarily imply biological independence.

Despite methodological differences (the UK Biobank elicited parental family history), our findings are consistent with two recent reports from the UK Biobank [6,7] demonstrating that polygenic risk and family history exert independent and additive effects for CHD risk assessment.

We recognize some limitations in our study. First, cohort participants were all members of KPNC, therefore findings may not fully generalize to uninsured populations. Second, the majority of patients were of European descent. Third, CARDIO inCode-Score® CHD PRS is not perfectly calibrated for ethnicities other than European. Fourth, we did not have information on whether the family history was premature or not (i.e., occurring at age <65 years if female or <55 if male), or number of affected relatives. Fifth, we relied on ICD and procedure codes without adjudication, although prior studies demonstrate their validity [4,5]. Finally, we did not have genetic assessment of familial hypercholesterolemia (FH) and it is possible that some of the increased risk of those with positive family history may have been driven by FH genes.

In conclusion, PRS and family history CHD were positively correlated, and both independently contribute to risk of incident CHD. Second, high PRS was associated with similar increased CHD risk in persons with and without family history CHD. A key finding of this study is therefore the fact that PRS adds to risk information among individuals with negative family history. Third, the joint effect of positive family history CHD and high PRS was well above 2. These results have important clinical implications because relying solely on self-reported family history is insufficient to fully characterize the genetic contribution to CHD and thus the use of PRS could complement traditional risk algorithms particularly among subjects with no known family history of CHD.

## Disclosure statement

Dr. Iribarren received a research grant from GEN inCode Plc.

Dr. Elosua is a member of the scientific advisory board of GEN inCode Plc and inventor of a patent based on the CARDIO inCode-Score® CHD Polygenic Risk Score (PRS) granted to and owned by GENinCode Plc. The remaining authors have nothing to disclose.

## Funding statement

This study was funded by a grant from GEN inCode, Plc.

## CRediT authorship contribution statement

**Carlos Iribarren:** Writing – original draft, Supervision, Resources, Project administration, Methodology, Funding acquisition, Conceptualization. **Meng Lu:** Formal analysis, Data curation. **Roberto Elosua:** Writing – review & editing. **Martha Gulati:** Writing – review & editing. **Nathan D. Wong:** Writing – review & editing. **Jamal S. Rana:** Writing – review & editing.

## Declaration of competing interest

The authors declare the following financial interests/personal relationships which may be considered as potential competing interests:

Carlos Iribarren, MD, MPH, PhD reports financial support was provided by Gen inCODE, Plc. Carlos Iribarren, MD, MPH, PhD reports a relationship with Gen inCODE, Plc that includes: funding grants. No other activities If there are other authors, they declare that they have no known competing financial interests or personal relationships that could have appeared to influence the work reported in this paper.
